# Evaluation of Facial Proportions, Landmarks Relationships With Facial and Dental Midlines, and Smile Framework

**DOI:** 10.1002/cre2.70164

**Published:** 2025-06-27

**Authors:** Zahra Bagheri, Vahid Mollabashi, Mohammad Mahdi Maleki, Behnaz Alafchi

**Affiliations:** ^1^ Department of Prosthetic Dental Implants Research Centre, Dental School Hamadan University of Medical Sciences Hamadan Iran; ^2^ Department of Orthodontics Dental School, Dental Research Centre Hamadan University of Medical Sciences Hamadan Iran; ^3^ Dental Implants Research Centre Hamadan University of Medical Sciences Hamadan Iran; ^4^ Student Research Committee Hamadan University of Medical Sciences Hamadan Iran; ^5^ Department of Biostatistics Hamadan University of Medical Sciences Hamadan Iran

**Keywords:** dental midlines, facial midline, facial proportions, smile framework

## Abstract

**Objective:**

Facial features play a key role in determining human attractiveness. Facial beauty is characterized by harmonious proportions and symmetry, essential for successful aesthetic treatments in oral rehabilitation. This study examines relationships between oral and facial midlines and key anatomical landmarks, as well as evaluates the smile framework and dental/facial proportions.

**Materials and Methods:**

A total of 637 males and females with an average age of 22.13 years (ranging from 18 to 40) were examined. Each individual had three digital images taken: a full‐face photo at rest, a forced smile photo, and an intraoral photo. For each patient, three levels of aesthetic analysis, from macro to micro aesthetics, were conducted. Initially, transverse and vertical facial proportions and their relationships with the golden ratio were assessed. Next, the relationships between predetermined important facial landmarks and the oral and facial midlines were evaluated, and finally, the patient's smile framework was analyzed. Data analysis was performed via IBM SPSS Statistics (v24.0) and appropriate statistical tests.

**Results:**

The studied population had a consonant smile arc, upward upper lip curvature, medium smile line with exposure up to the first molar, and square‐shaped teeth. Order of landmarks relative to facial midline from left to right includes the following: nasion, nose tip, philtrum, mouth midline, and dental midline. Furthermore, five out of seven vertical facial proportions and all transverse facial proportions closely align with the divine ratio (1.618), with significant gender‐specific differences.

**Conclusion:**

Integration of these aesthetic standards are necessary to ensure satisfactory and predictable outcomes in treatments and can be used at macroesthetic, miniesthetic, and microesthetic levels by maxillofacial surgeons, prosthodontists, orthodontists, and restorative specialists. According to impact of race, sex, and age on these factors, conducting similar studies in different populations is recommended to achieve more precise and aesthetically pleasing treatments tailored to each community.

## 1 Introduction

The term “aesthetics” refers to beauty, naturalness, and a youthful appearance relative to age. Aesthetics motivate patients to seek dental treatment. The face is a key feature in determining human physical attractiveness. Symmetry is one of the factors contributing to facial harmony and determines the aesthetic success of our dental procedures in aesthetic dentistry (Eskelsen et al. 2009; Kahn et al. 2024). Symmetry refers to the matching in size, shape, and relative position of parts on opposite sides around a center or axis, known as the “midline.” All aesthetic deviations revolve around the midline (Iwanaga et al. 2016; Rakhshan et al. 2024; Padovezi et al. 2025). Facial harmony depends on the interrelationship of facial components such as the nose, eyes, lips, and chin. Its application in restorative or rehabilitative procedures can determine the aesthetic success of the treatment (Eskelsen et al. 2009). An individual's facial beauty is also associated with harmonious proportions. Ideal proportions have a direct correlation with so‐called divine proportions, with the most significant ratio being 1:1.618 (Borelli and Berneburg 2010; Pancherz et al. 2010; Husein et al. 2010).

Various facial landmarks in the middle third of the face, such as the midpoint between the pupils, the nasion point, the tip of the nose, the philtrum tip, and the chin, are used to determine the facial and dental midlines. Some studies prefer the use of intraoral points such as the incisive papilla to determine the maxillary dental midline. The alignment of the dental midline with either the facial midline or the mouth midline has been debated in other studies (Farahani et al. 2019). Some believe that since patients tend to associate their dental midline with proximal structures rather than with distant anatomical features, aligning the dental midline with the mouth midline is sufficient (Cunha et al. 2023). Clinical studies have limited the deviation of dental midlines from the facial midline to approximately 2‐3 mm (Rosenstiel and Rashid 2002; Cardash et al. 2003). According to Lombardi's suggestion, the dental midline is located at the most stable position on the face (Chahuara‐Ramírez and Arriola‐Guillén 2024). Farka defines the facial midline as a line passing through three anatomical landmarks: the nasion, subnasale, and gnathion or menton (Farkas 1981). However, this definition lacks clarity and is prone to subjectivity and reproducibility issues for research purposes. Currently, glossaries such as the Glossary of Prosthodontic Terms, the American Association of Orthodontists' glossary, and the American Academy of Facial Plastic and Reconstructive Surgery's glossary do not provide specific definitions for facial and dental midlines (Committee AoDPN 1987).

In addition to their aesthetic relevance, facial landmarks and midline relationships have long been studied in anthropometric and craniofacial research. Understanding the relative positions of these features can provide insights into population‐based facial proportions, biological variation, and normative reference standards. Such measurements are frequently used in comparative studies across different ethnic or demographic groups, and contribute not only to aesthetic analysis but also to orthodontic diagnosis and surgical planning. Despite aesthetic goals being the primary concern in many dental settings, appreciating the structural and measurable relationships between facial and dental landmarks offers a more comprehensive understanding of the harmony and function of the smile. Therefore, incorporating both aesthetic judgment and anatomical evaluation can enhance treatment outcomes and support cross‐population research (Milošević et al. 2008; Edler et al. 2006; Proffit et al. 2019).

For greater aesthetics, the maxillary dental midline should align with the facial midline owing to the visibility of the maxillary anterior teeth during smiling, rather than the mandibular midline. While it is desirable for the facial, maxillary, and mandibular midlines to align, it is not necessary (Eskelsen et al. 2009). Various authors have described the ideal characteristics of a smile (Machado 2014; de Deus Tupinambá Rodrigues et al. 2009; Khan et al. 2020), although deviations from aesthetic dimensions have been observed in different populations (Chahuara‐Ramírez and Arriola‐Guillén 2024; Melo et al. 2019; Musa et al. 2023). Methods for evaluating the smile include photographs (Silva et al. 2013), videos (Dindaroğlu et al. 2016), and 3D stereophotogrammetric images (Duran et al. 2017).

There are two types of smiles: spontaneous and forced. The former is involuntary and can be considered an expression of genuine emotions, whereas the latter is repeatable and thus serves as a reference position (Ackerman et al. 1998).

In facial analysis, we have several transverse reference lines (the interpupillary line and other lines parallel to it) and a vertical midline located at two anatomical points: the nasion and the philtrum (Silva et al. 2013). The smile arc can be defined as the relationship between the curvature of the maxillary incisal edges and the curvature of the lower lip in a posed smile, which may be consonant or non‐consonant (Sabbah 2022).

Practitioners have noted an increasing demand for treatments based on aesthetic principles. Consequently, orthodontists, maxillofacial surgeons, and restorative and prosthodontic specialists must have a comprehensive understanding of the quantitative and objective facial features widely considered attractive and beautiful (Milošević et al. 2008). This is particularly true in orthodontic treatment, where the goals at the end of treatment include not only functional stability but also improved facial aesthetics, which remains one of the most common reasons for seeking orthodontic treatment today (Edler et al. 2006; Proffit et al. 2019).

This study aims to examine the relationship between facial and dental midlines and anatomical landmarks, as well as evaluate the smile framework and facial proportions.

## 2 Materials and Methods

### 2.1 Sample

The required sample size was calculated based on prior studies (Farahani et al. 2019; Kurian et al. 2018), using α = 0.05 and power = 80%, yielding a minimum of 49 participants. However, to enhance the robustness and generalizability of results, a larger sample was included.

A total of 750 male and female patients, randomly selected from an area in Asia, were included. Digital photographs of each subject's entire face at rest and in forced smiles, as well as intraoral photographs, were taken with the subject seated in a chair. A digital camera (Nikon COOLPIX P610, Nikon Corporation, Japan) was mounted on a tripod with standard focus and a fixed distance of 1.5 meters from the subject, with constant lighting conditions. For frontal facial photographs, the focus point and center of the image were the intersection between the Frankfort horizontal plane and the facial midline, whereas for intraoral photographs, they were the incisal plane and dental midline. The camera lens height was adjusted to the eye level of each subject, with the head position evaluated in its natural transverse and vertical axes without rotation. Imaging software (Adobe Photoshop CS, Adobe Systems Inc., San Jose, California, USA) was used for image analysis.

The inclusion criteria for the study participants were as follows:

1. Age between 18 and 40 years old.
2. No history of orthodontic treatment.
3. Presence of natural anterior teeth.
4. No anterior maxillary teeth were replaced via any prosthesis or restoration.
5. No history of congenital diseases or trauma affecting facial form or appearance.
6. Ability to comprehend and provide written informed consent.

The exclusion criteria for photographs included the following:

1. Images with rotated head position (non‐frontal view)
2. Visible asymmetry including the eyes
3. Incorrect clinical marking
4. Poor resolution images

Ultimately, photographs of 637 patients (350 females and 287 males) with an average age of 22.13 ± 4.29 years old (ranging from 18 to 40) were approved, and the following analyses were conducted, with methods for each detailed below:

### 2.2 Facial Proportions

**FIGURE 1:**
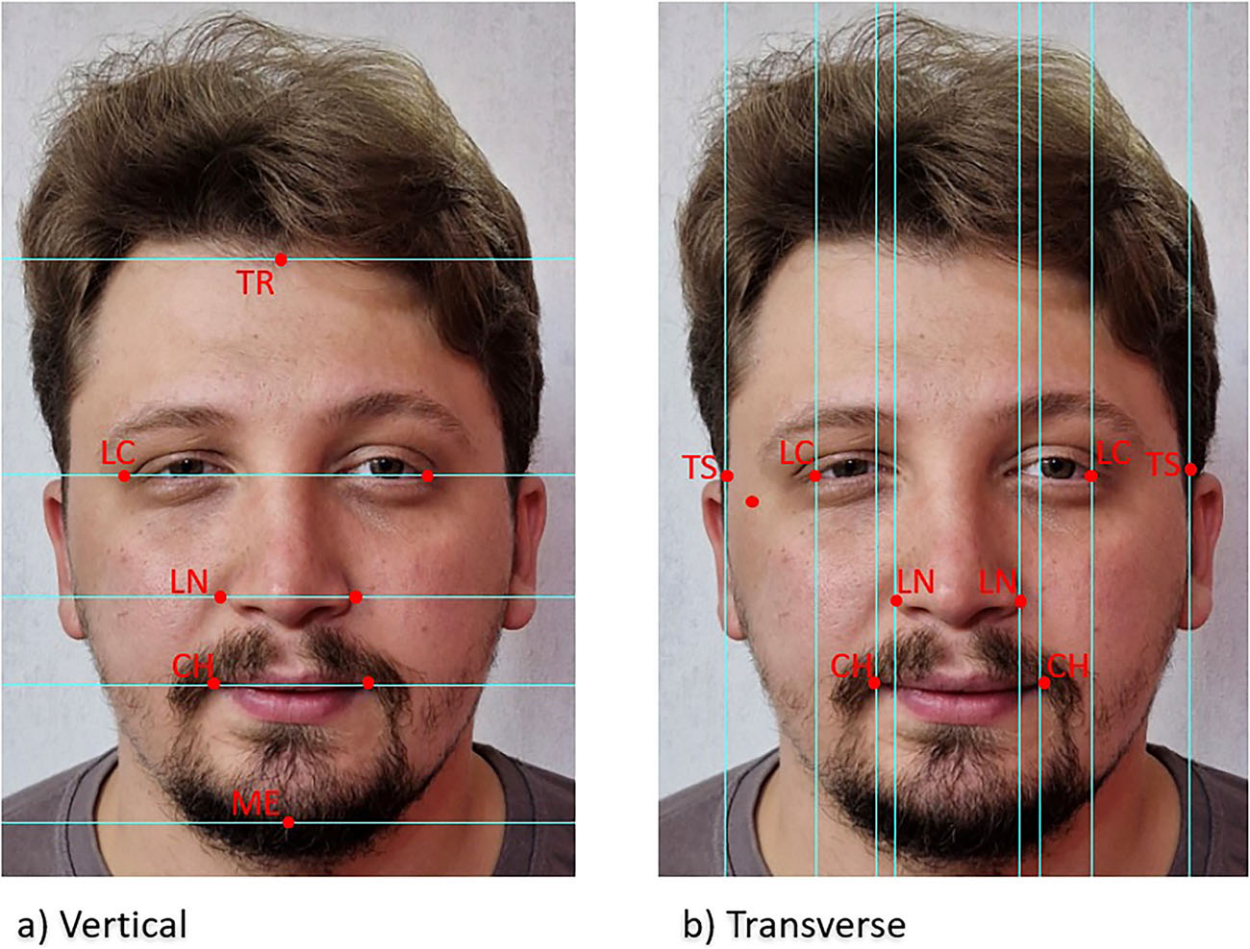
Photographic points and vertical (a) and transverse (b) linear measurements used in the study.

The TR (top of the forehead at the hairline), TS (temporal soft tissue above the ear at the supraorbital ridges), LC (outer canthus of the eye), LN (lateral edge of the nose), CH (corner of the mouth), and ME (soft tissue menton) points were identified on each photograph, with CH, TS, LC, and LN marked on both sides (Figure 1). The vertical distances TR‐ME, LC‐ME, TR‐LC, TR‐LN, LN‐ME, LC‐LN, LC‐CH, CH‐ME, and LN‐CH and the transverse distances between the corresponding points CH, LC, and TS on both sides were calculated (Khan et al. 2016) (Figure 1).

### 2.3 Landmark Relationships With Facial and Dental Midlines

Standard definitions for landmarks were used for the study. The external canthus was defined as the external angle formed by the junction of the upper and lower eyelids, nasion as the point at the midline root of the nose and nasofrontal suture, philtrum as the vertical groove on the midline of the upper lip, commissure as the point or line of union between two anatomical parts (lips), and pronasale as the most prominent point of the nose tip. These definitions were used for all clinical markings and digital “beauty frame” constructions. Owing to the near impossibility of determining the facial midline in static and dynamic movements, a rectangular frame was used to define the facial midline. This frame was defined as the area on the human face where aesthetic landmarks such as midlines, canthus, and smile parameters are perceptually and objectively validated.

**FIGURE 2:**
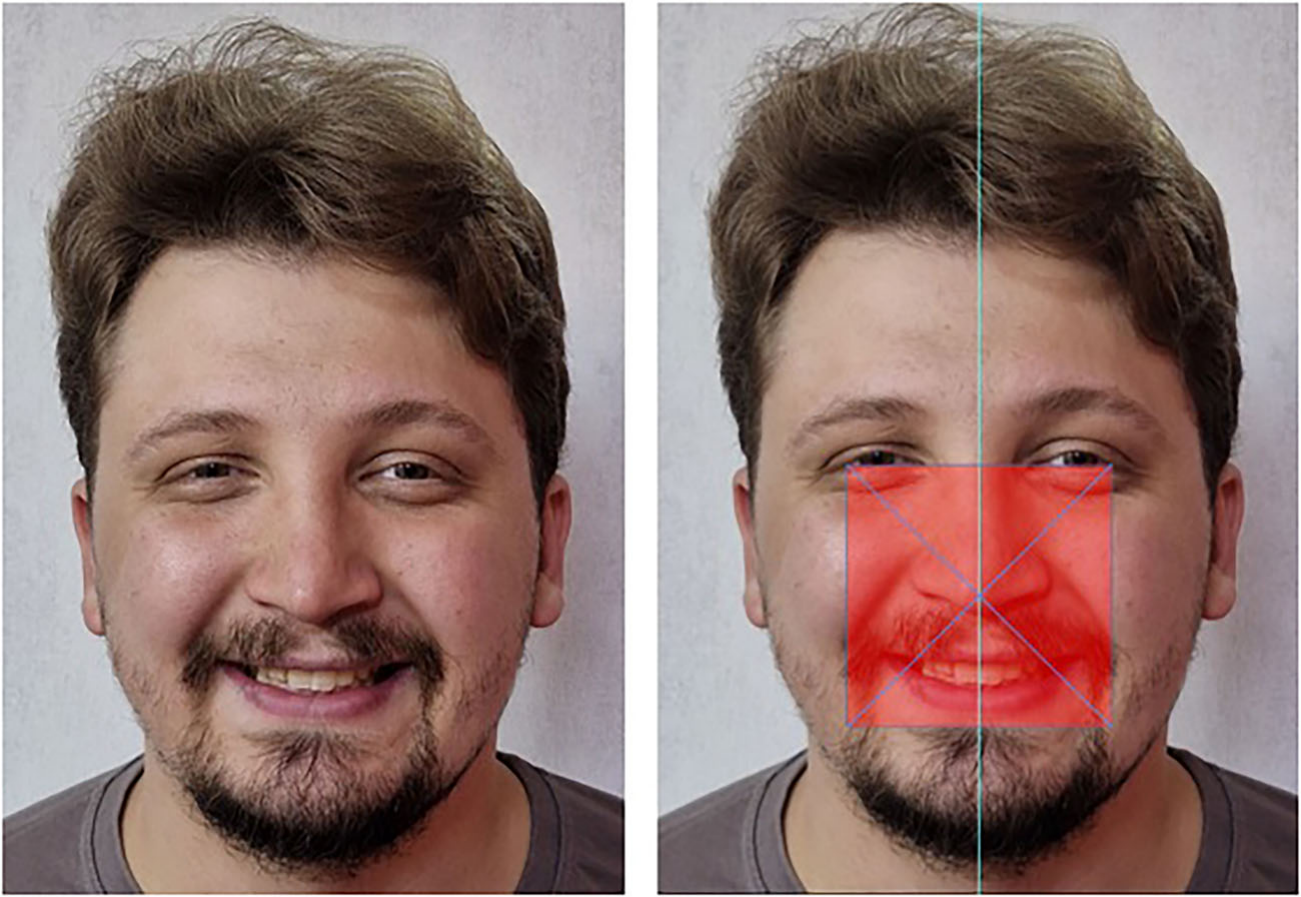
Sample photo to illustrate the concept of a beautiful frame used to achieve objectivity and reliability.

The upper boundary was marked by a line drawn from the external canthus of one eye to the external canthus of the other eye, helping to neutralize any slight head rotation along the sagittal axis. Therefore, subjects with ocular asymmetry were excluded from analysis using this frame. Two lateral boundaries were drawn perpendicular to the external canthus of each eye, parallel to each other. The lower boundary was parallel to the line drawn at the lowest border of the lower lip, completing the four sides of the frame (Figure 2).

Obtaining the midline of part of the face included in this aesthetic frame is more crucial than obtaining the actual midline of the entire face, as it excludes tissues not related to the perception of the facial midline, such as the chin, buccal soft tissues, and forehead. The dynamic nature of the mandible, irregular hypertrophies of the buccinators and masseter muscles, and variable forehead size can potentially act as confounding variables in midline perception. In this study, three midlines were determined for each subject: the facial midline as the midline of the aesthetic frame, the dental midline as the vertical line through the cusp tip of the maxillary central incisors parallel to the vertical lines of the facial aesthetic frame, and the mouth midline as the line bisecting the distance between the mouth corners in a smiling pose. The relative facial midline value (RFV) and relative commissural midline value (RCV) are tools used to determine the relationship of landmarks to their respective midlines.

**FIGURE 3:**
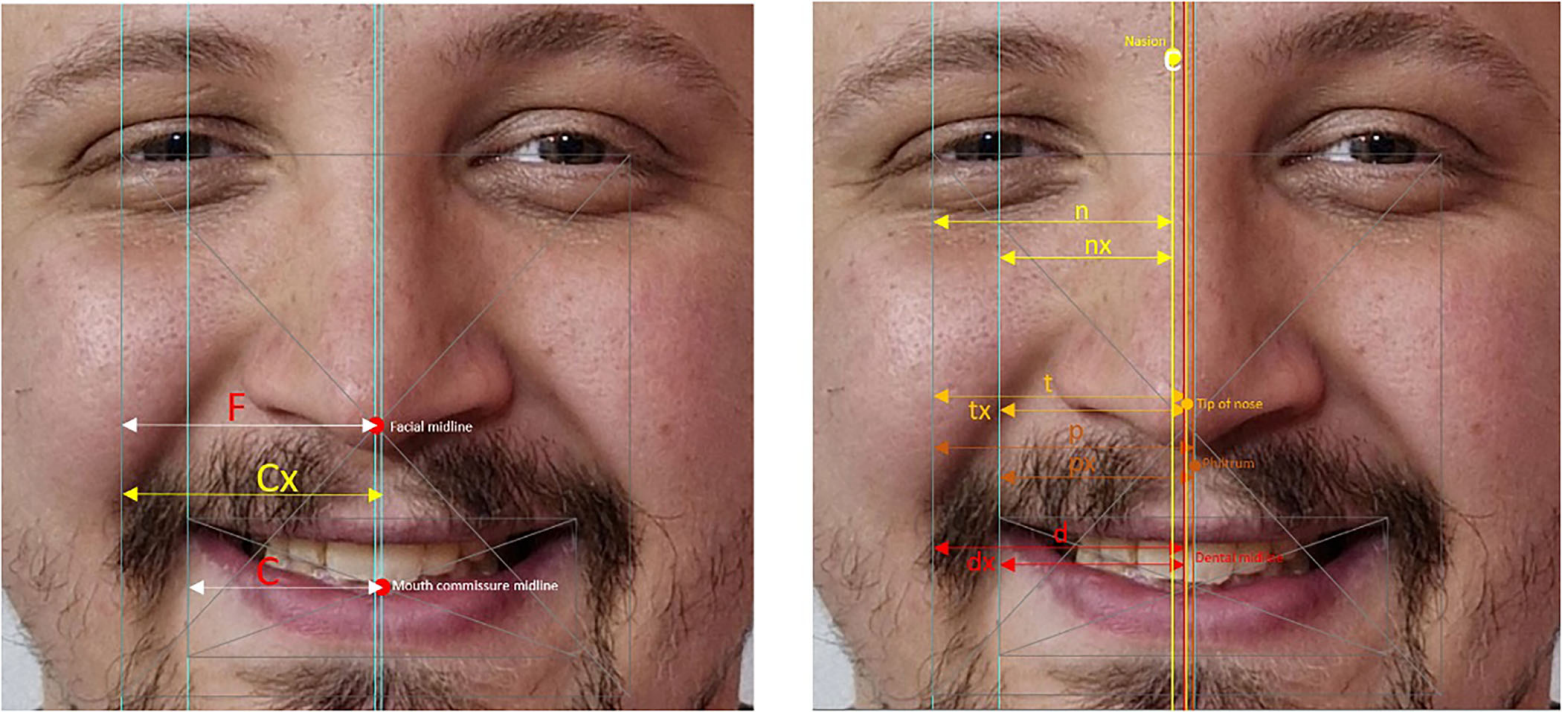
Details of beauty frame analysis: Method of determining the RFV and RCV values for each anatomic landmark. F: midline of the face/midline of the aesthetic frame; n: distance between the nasion and the lateral border of the aesthetic frame; t: distance between the tip of the nose and the lateral border of the aesthetic frame; p: distance between the tip of the philtrum and the lateral border of the aesthetic frame; d: distance between the dental midline and the lateral border of the aesthetic frame; C: midline of the oral commissures; Cx: distance between the midline of the commissures and the lateral border of the aesthetic frame; nx: distance between the nasion and the oral commissures; tx: distance between the tip of nose and the oral commissures; px: distance between the tip of philtrum and the oral commissures; dx: distance between the dental midline and the oral commissures.

The facial midline was created by halving the distance between the two lateral borders of the frame, and then three vertical lines were drawn through each anatomical landmark (nasion, nose tip, and philtrum). The fourth line was drawn along the subject's existing dental midline as defined above. The RFV was defined as the relative distance of a landmark to the facial midline. The measured distance from the lateral frame border to the established facial midline was considered a constant “F.” The distance from the lateral frame border to the nasion was a variable “n.” RFV was obtained by dividing n by F. Similarly, the RFVs for the other three landmarks—nose tip (t), philtrum tip (p), and dental midline (d)—were obtained by dividing them by the constant F. Numerical values for n/F, t/F, p/F, and d/F were thus derived (Figure 3).

The RCV was defined as the relative distance of a landmark to the mouth commissure midline (center of the mouth). The measured distance from the midpoint of the line between the commissures to the right canthus was considered a constant “C.” The values for the philtrum (px) and dental midline (dx) were obtained. The RCV was then derived by dividing nx/C, tx/C, px/C, and dx/C. The distance from the lateral aesthetic frame border to the midpoint of the commissures was described as a variable Cx. The relationship between the commissure midline and facial midline was obtained by dividing Cx/F (Figure 3). The primary reason for using RFV and RCV was to establish a proportional relationship between the landmarks and their respective midlines, ensuring a standardized common denominator for all landmarks in the aesthetic frame and negating the need to match the image size to the subject's face. The relationships for landmark ratios for both midlines were RFV1 and RCV1: ratios of nasion to facial and commissural midlines. RFV2 and RCV2: ratios of nose tip to facial and commissural midlines. RFV3 and RCV3: ratios of the philtrum tip to the facial and commissural midlines. RFV4 and RCV4: ratios of the dental midline to the facial and commissural midlines. RFV5: ratio of the commissural midline to the facial midline. Thus, in perfect symmetry, all five RFVs and four RCVs are equal to 1. The right lateral frame or commissure border was chosen for assessment. Therefore, an RFV or RCV less than 1 would indicate deviation to the right, and an RFV or RCV greater than 1 would indicate deviation to the left. If a line drawn through one anatomical landmark coincided with another, the same RFV or RCV value was recorded for both. If an anatomical landmark coincided with the facial or commissural midline, its RFV or RCV value was assigned a value of 1 (Bidra et al. 2009).

### 2.4 Smile Arc

**FIGURE 4:**
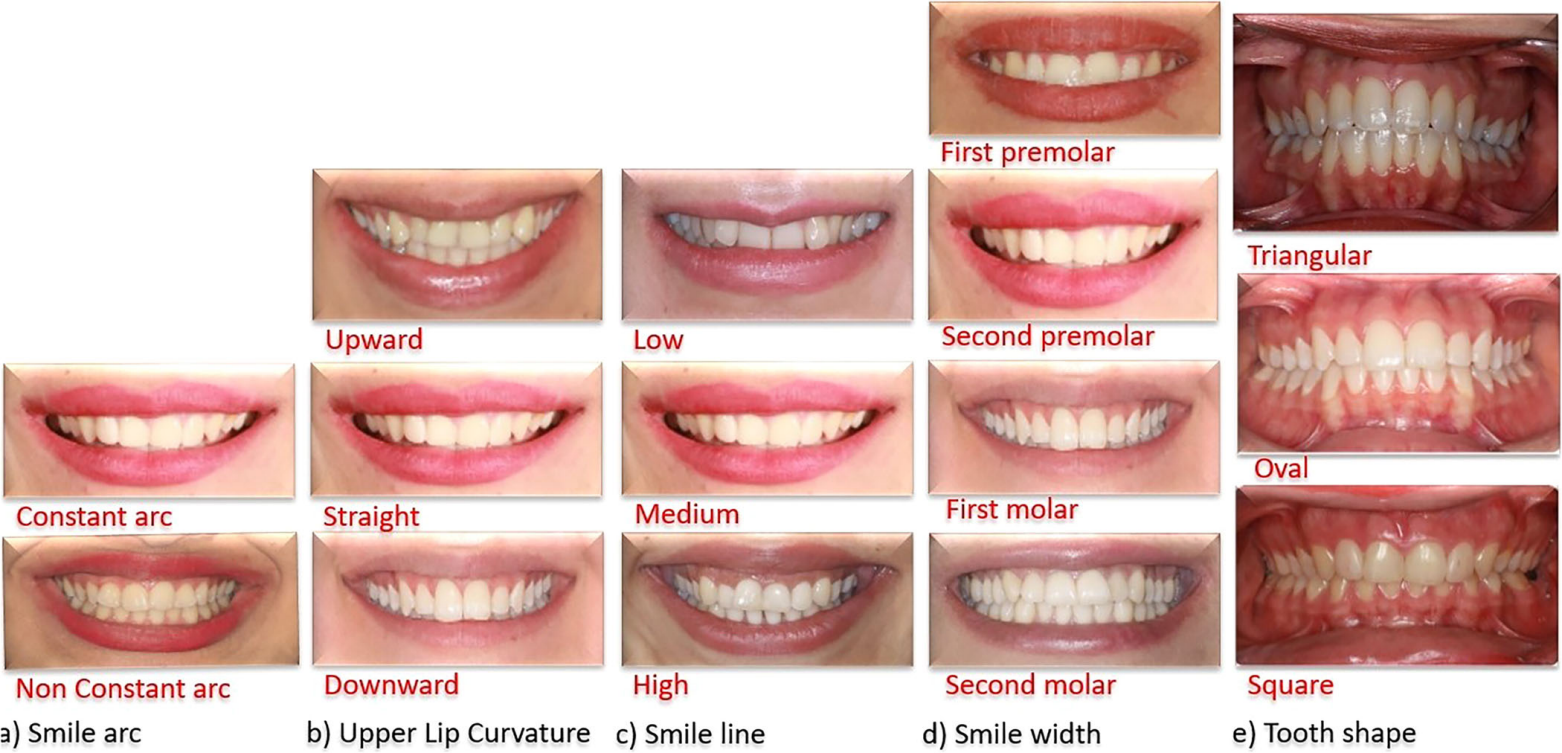
Smile frame details: (a) Smile arc, (b) Upper Lip Curvature, (c) Smile line, (d) Smile width and (e) Tooth shape.

The smile arc is defined as the relationship between the curvature of the maxillary incisors and canines and the curvature of the lower lip in a posed smile, which can be consonant or non‐consonant (Figure 4a).

### 2.5 Upper Lip Curvature

The upper lip curvature can be classified as upwards, straight, or downwards on the basis of the position of the mouth corners relative to the center of the lower lip margin (Figure 4b).

### 2.6 Smile Line

The smile line is classified as high if it reveals the entire clinical crown with a variable band of continuous gingiva, medium if it reveals 75%–100% of the clinical crown and only the interdental papilla, and low if it shows less than 75% of the clinical crown (Figure 4c).

### 2.7 Smile Width

Smile width is determined by the number of teeth exposed during smiling (Figure 4d).

### 2.8 Tooth Shape

The relationship between the shape and ratio of the right central incisor (DP) was calculated via the formula DP% = (length (mm)/maximum width (mm)) × 100. Teeth are classified as triangular (DP < 43%), oval (43% < DP < 57%), or square (DP > 57%) (Figure 4e) (Melo et al. 2020).

### 2.9 Tooth Size

The width and length of the teeth are measured, and the width‐to‐length ratio is calculated and compared with the golden ratio of 1.618 (Kalia 2020).

### 2.10 Statistical Analysis

Data analysis was conducted via IBM SPSS Statistics software (version 24.0; IBM Corp) and appropriate statistical tests. To ensure the reliability and validity of the results, the survey and data analysis were repeated twice. Descriptive statistics were calculated for each parameter in the overall sample and stratified by sex, with sex serving as the primary independent variable to investigate potential sexual dimorphism.

To determine the associations between various qualitative variables and sex, the chi‐square test was used. The Fisher exact test was applied when contingency tables had more than 33% of cells with expected frequencies below five cases, allowing for the assessment of relationships between two dichotomous variables. The Mann‐Whitney U‐test, a nonparametric test, was used to compare the distributions of quantitative variables between two genders.

## 3 Results

### 3.1 Facial Divine Proportion

**FIGURE 5:**
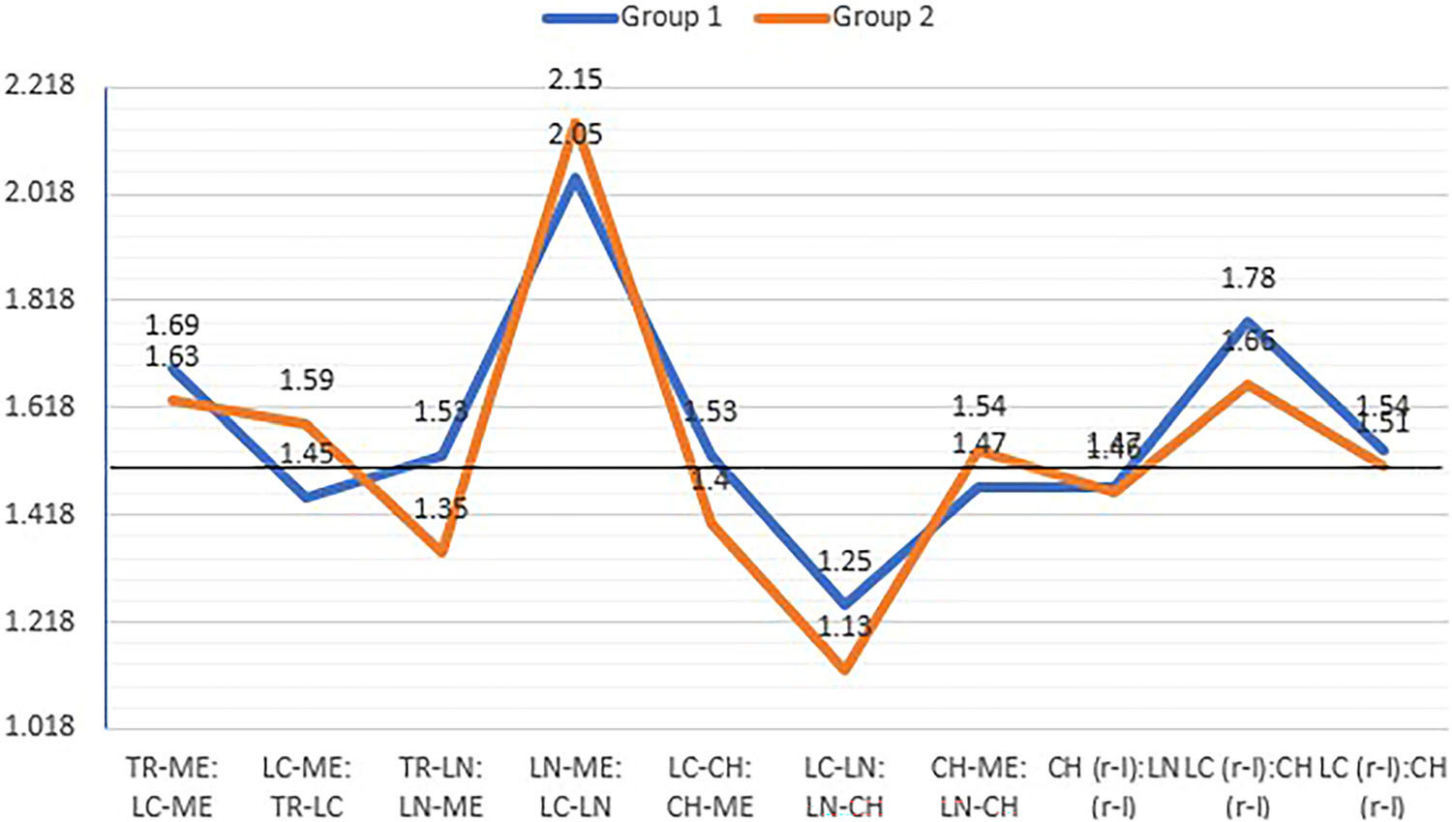
Deviation of the vertical and transverse facial proportions from divine proportions in both groups.

To assess the vertical and transverse facial proportions, the distances between identified points were measured, and the ratios in Table 1 were calculated. The deviation from the golden ratio of 1.618 was also examined (Petekkaya et al. 2021). In Group 1 (females), the vertical ratios were TR‐ME: LC‐ME, LC‐ME: TR‐LC, TR‐LN: LN‐ME, CH‐ME: LN‐CH, LC‐CH: CH‐ME, and all transverse ratios, and in Group 2 (males), the vertical ratios were TR‐ME: LC‐ME, LC‐ME: TR‐LC, CH‐ME: LN‐CH, and again all transverse ratios were very close to the divine ratio (Table 1, Figure 5).

**TABLE 1:** Comparison of various facial proportions across the study groups.

Facial proportions		Group I (females)		Group II (males)		*P*
		Mean ± SD	% value (considering 1.618 = 100%)	Mean ± SD	% value (considering 1.618 = 100%)	
Vertical proportions	TR‑ME: LC‑ME	1.69 ± 0.06	105	1.63 ± 0.06	100.90	0.001
	LC‑ME: TR‑LC	1.45 ± 0.14	90.16	1.59 ± 0.16	98.73	0.001
	TR‑LN: LN‑ME	1.53 ± 0.16	94.73	1.35 ± 0.16	83.78	0.001
	LN‑ME: LC‑LN	2.05 ± 0.28	126.92	2.15 ± 1.01	133.09	0.001
	LC‑CH: CH‑ME	1.53 ± 0.12	94.83	1.40 ± 0.16	86.57	0.001
	LC‑LN: LN‑CH	1.25 ± 0.55	77.32	1.13 ± 0.22	70.05	0.009
	CH‑ME: LN‑CH	1.47 ± 0.32	90.90	1.54 ± 0.24	95.42	0.001
Transverse proportions	CH (r‐l): LN (r‐l)	1.47 ± 0.13	90.94	1.46 ± 0.46	90.44	0.001
	LC (r‐l): CH (r‐l)	1.78 ± 0.27	110.41	1.66 ± 0.18	103.12	0.001
	TS (r‐l): LC (r‐l)	1.54 ± 0.96	95.37	1.51 ± 0.25	93.54	0.409

### 3.2 Landmark Relationships With Facial and Dental Midlines

To assess the reliability of the RFV and RCV measurements, these values were measured twice. The intraclass correlation coefficients (ICCs) for reliability analysis indicated acceptable reliability for all the values, demonstrating the high consistency and accuracy of the measurements. All the ICCs were statistically significant beyond the alpha level of 0.001 (Table 2).

**TABLE 2:** Reliability analysis of the RCV and RFV data.

Item Pair			Reliability
RVF	Nasion	RFV11 ‐ RFV12	0.87
	Tip of nose	RFV21 ‐ RFV22	0.89
	Tip of philtrum	RFV31 ‐ RFV32	0.93
	Dental midline	RFV41 ‐ RFV42	0.95
	Midline of commissures	RFV51 ‐ RFV52	0.88
RCV	Nasion	RCV11 ‐ RCV12	0.89
	Tip of nose	RCV21 ‐ RCV22	0.92
	Tip of philtrum	RCV31 ‐ RCV32	0.94
	Dental midline	RCV41 ‐ RCV42	0.95

**FIGURE 6:**
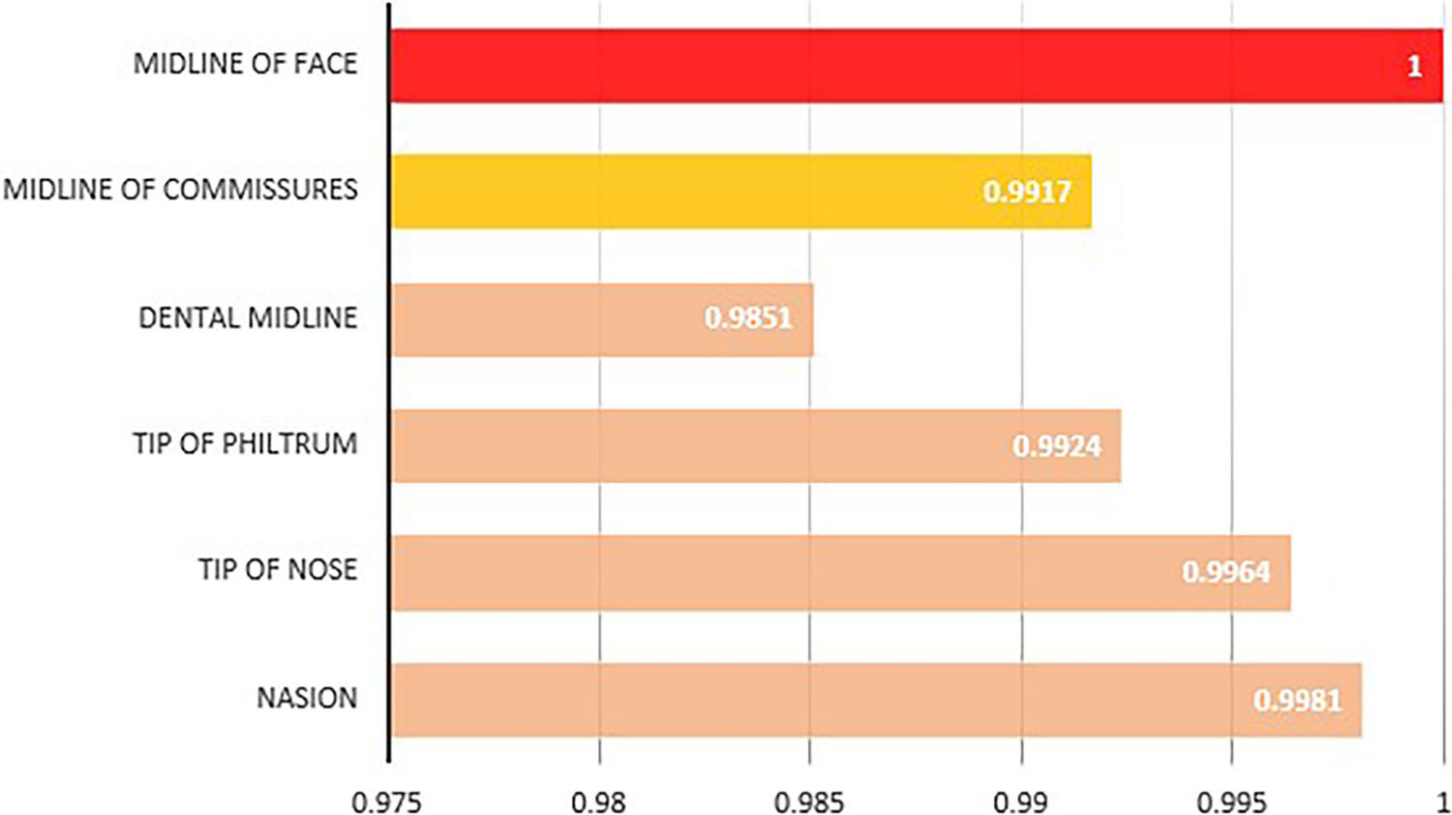
Hierarchical relationship of anatomic landmarks with the midline of the face and the midline of commissures.

On average, from left to right, the nasion, nose tip, philtrum, mouth midline, and dental midline were positioned, on the right of the facial midline. For the RCV, the nose tip, philtrum, and nasion were on the left, and the dental midline was on the right of the mouth midline. Analysis revealed that the difference between the mean ratio of each anatomical landmark and the facial and mouth midlines was statistically significant (*p* < 0.001). Except for RFV3 (philtrum deviation from the facial midline), no significant differences were observed in the other measurements between males and females (Table 3, Figure 6).

**TABLE 3:** Various RCV and RFV values and their comparisons among different genders.

Landmark		Total		Sex				
				Female		Male		*P*
		Mean	Standard Deviation	Mean	Standard Deviation	Mean	Standard Deviation	
RVF1	Nasion	0.9981	0.08555	0.9994	0.0888	0.9943	0.0755	0.334
RVF2	Tip of nose	0.9964	0.07881	0.9950	0.08887	1.0007	0.03707	0.102
RVF3	Tip of philtrum	0.9924	0.07127	0.9912	0.07960	0.9957	0.03851	0.050
RVF4	Dental midline	0.9851	0.08949	0.9853	0.09340	0.9844	0.07767	0.173
RVF5	Midline of commissures	0.9917	0.07433	0.9910	0.08309	0.9937	0.03986	0.330
RCV1	Nasion	1.0153	0.08463	1.0203	0.06687	1.0007	0.12144	0.394
RCV2	Tip of nose	1.0039	0.09207	1.0058	0.07869	0.9985	0.12308	0.990
RCV3	Tip of philtrum	0.9987	0.06535	1.0008	0.03555	0.9927	0.11392	0.497
RCV4	Dental midline	0.9902	0.11492	0.9960	0.03675	0.9737	0.21753	0.200

To compare the nasion and nose tips as well as the philtrum and dental midline, Pearson correlation analysis was performed to determine their significance. Table 4 shows that RCV1 correlated significantly with RCV2 and that RCV3 correlated with RCV4 at the 0.01 level (2‐tailed), indicating similar values.

**TABLE 4:** Pearson correlation coefficient between RCV1 ‐ RCV2 and RCV4 ‐ RCV3 to show the reversal of the hierarchy.

		RCV1	RCV2
RCV1	Pearson correlation	1	0.621
	Significance (2‐tailed)		0.001
	N	337	337
RCV2	Pearson correlation	0.621	1
	Significance (2‐tailed)	0.001	
	N	337	337

		RCV3	RCV4
RCV3	Pearson correlation	1	0.907
	Significance (2‐tailed)		0.001
	N	337	337
RCV4	Pearson correlation	0.907	1
	Significance (2‐tailed)	0.001	
	N	337	337

### 3.3 Smile

The smile arc was a consonant in 82.8% of individuals, with 83.15% of females and 84.10% of males having a consonant smile arc (*p* > 0.05). The upper lip curvature was upwards in 53.7%, straight in 27.9%, and downwards in 18.4% of individuals. An examination of the upper lip curvature revealed that 14.5% of the females and 6.5% of the males presented an upwards curvature, 6.5% of the females and 21.4% of the males presented a straight curvature, and 4.7% of the females and 13.6% of the males presented a downwards curvature (*p* > 0.05). The smile line was high in 25.8%, medium in 58.2%, and low in 16% of the individuals. Among the females, 16.84% had a high smile line, 55.93% had a medium smile line, and 26.82% had a low smile line. Among the males, 12.79% had a high smile line, 64.34% had a medium smile line, and 22.87% had a low smile line (*p* > 0.05). The smile width was most frequently (49.3%) up to the first molar, followed by the second premolar (37.7%), first premolar (10.1%), and second molar (3%). Among females, 47.57%, 40.43%, 9.57%, and 2.43% had smile widths up to the first molar, second premolar, first premolar, and second molar, respectively. Among males, 53.88%, 29.85%, 11.63%, and 4.65% had smile widths up to the first molar, second premolar, first premolar, and second molar, respectively (*p* > 0.05). Finally, tooth shape was analyzed, with 57.7%, 39%, and 3.3% of individuals having square, oval, and triangular teeth, respectively. Among females, 60.75% had square teeth, 36.83% had oval teeth, and 1.8% had triangular teeth. Among the males, 48.83%, 45.31%, and 5.86% had square, oval, and triangular teeth, respectively. The mean central incisor length and width were 100.72 ± 83.54 mm and 85.68 ± 70.88 mm, respectively, with a mean width‐to‐length ratio of 1.1845 ± 0.14123, which was 73.20% of the golden ratio of 1.618 (Table 5).

**TABLE 5:** Analysis of smile framework parameters in the total population and by sex group.

			Total	Sex		
				Female	Male	*p*
Smile	Constant	N	527	291	234	0.735
		Percent	82.8	82	81.61	
	Non constant	N	58	59	53	
		Percent	110	18	18.39	
Curve of upper lip	Up	N	342	185	161	0.805
		Percent	53.7	52.80	56.32	
	Straight	N	178	101	73	
		Percent	27.9	28.80	25.29	
	Down	N	117	64	53	
		Percent	18.4	18.40	18.39	
Smile line	High	N	164	94	66	0.372
		Percent	25.8	26.80	22.99	
	Medium	N	371	196	185	
		Percent	58.2	56	64.37	
	Low	N	102	60	36	
		Percent	16	17.20	12.64	
Smile width	First premolar	N	64	34	33	0.288
		Percent	10.1	9.6	11.49	
	Second premolar	N	240	141	86	
		Percent	37.7	40.4	29.89	
	First molar	N	314	167	155	
		Percent	49.3	47.6	54.02	
	Second molar	N	19	8	13	
		Percent	3.0	2.4	4.60	
Tooth shape	Triangle	N	21	8	20	0.078
		Percent	3.3	2.4	6.90	
	Oval	N	248	129	129	
		Percent	38.9	36.80	44.83	
	Square	N	368	213	138	
		Percent	57.6	60.80	48.28	
Tooth size	Width	Mean ± SD	85.6795 ± 70.88140	87.2400 ± 71.14297	81.1954 ± 70.33926	0.245
	Height	Mean ± SD	100.7172 ± 83.54130	101.7540 ± 83.03876	97.7379 ± 85.38486	0.882
	Width/height	Mean ± SD	1.1845 ± 0.14123	1.1780 ± 0.12939	1.2029 ± 0.17026	0.014

## 4 Discussion

With respect to facial proportions, Ricketts conducted the first study on the application of mathematical ratios in aesthetics, suggesting the use of quantitative proportions instead of subjective perceptions. He used the golden ratio for facial proportion assessment but included only a small sample of 10 frontal photographs, resulting in low validity of the findings (Ricketts 1982). In our study, the vertical ratios TR‐ME: LC‐ME, LC‐ME: TR‐LC, TR‐LN: LN‐ME, CH‐ME: LN‐CH, and LC‐CH: CH‐ME, and all transverse ratios in females and the vertical ratios TR‐ME: LC‐ME, LC‐ME: TR‐LC, and CH‐ME: LN‐CH, and all transverse ratios in males were very close to the divine ratio. In Naseem Ahmad Khan et al. (Khan et al. 2016) and Mizumoto et al. (Mizumoto et al. 2009), five of the seven ratios (TR‐ME: LC‐ME, LC‐ME: TR‐LC, LC‐CH: CH‐ME, and LC‐LN: LN‐CH) in attractive females were close to the golden ratio, which supports our results. In our male group, three ratios were close to the golden ratio, indicating that male ratios deviate more from the golden ratio than do female ratio, as confirmed by Ahmad Khan et al. (Khan et al. 2016) and Mizumoto et al. (Mizumoto et al. 2009). Omotoso et al. (Omotoso et al. 2011) also reported differences in vertical ratios between males and females. The transverse ratios were closer to the divine ratio in both groups. A decrease in CH (r‐l):LN (r‐l) and TS (r‐l):LC (r‐l) and an increase in LC (r‐l):CH (r‐l) indicate a smaller mouth size. These results were also reported by Ahmad Khan et al. (Desai et al. 2009) and Mizumoto et al. (Mizumoto et al. 2009). On the basis of these observations, males' lower facial regions and females' upper facial regions occupy a larger portion of the vertical dimension, whereas females' eye and mouth widths occupy a smaller portion of the transverse dimension than males do. Farkas et al. reported that Americans, African‐Americans, Caucasians, Malaysians, Indians, Arabs, and Chinese people have different facial features influenced by race and ethnicity (Farkas et al. 2005).

In the examination of anatomical landmarks and facial and dental midlines, the smiling images of the subjects were used to ensure the visibility of the dental midline, with attention given to avoiding severely asymmetrical smiles. Anatomical landmarks were clinically marked on the samples for accurate identification, and marking errors from the photographs were reduced. Notably, the difficulty in identifying certain anatomical landmarks, such as the soft tissue nasion and nose tip, due to their inherent anatomical variations was anticipated, and the markings were cross‐verified by three reviewers to minimize errors. Exclusion criteria for samples and the use of an aesthetic frame instead of the entire photograph were implemented to reduce potential confounders. In this study, the mouth commissure midline was found to be close to the facial midline, indicating symmetrical placement of the mouth relative to the eyes. The dental midline also showed good alignment with the facial and mouth midlines, as supported by other studies (Bidra et al. 2009). The philtrum tip showed greater alignment with the mouth midline than with the facial midline, as noted in other studies (Rosenstiel and Rashid 2002; Cardash et al. 2003; Bidra et al. 2009). The nasion had the highest alignment with the facial midline, indicating its placement between the eyes. Pearson correlation analysis of the relationships among the nasion, nose tip, philtrum, and dental midline revealed that these anatomical points are similar and follow each other, with the nasion and nose tip similarity confirmed by other studies (Bidra et al. 2009). This was the first study to examine the similarity between the philtrum and the dental midline. Therefore, for aesthetic procedures in the smile area, the alignment of the philtrum with the mouth midline, and subsequently the dental midline, is important. For reconstructive procedures in the midface and nasal areas, the alignment of the nasion and nose tip with each other and the facial midline is more critical. Studies have indicated that sensitivity to midline discrepancies varies among different cultures, but most people value naturalness in reconstructions (Sadrhaghighi et al. 2017; Akinboboye et al. 2018).

Ethnicity and gender influence smile characteristics (Desai et al. 2009; Nold et al. 2014). In this study, the majority had a consonant smile arc and an upwards upper lip curvature. Other studies have also shown a consonant smile arc, although Maulik and Nanda's study rejected this finding (Desai et al. 2009; Nold et al. 2014; Maulik and Nanda 2007). Most studies agree with our finding of the upwards curvature of the upper lip (Melo et al. 2020), but studies in China reported a straight curvature (Del Monte et al. 2017; Liang et al. 2013), likely because of the different populations studied. The majority had a medium smile line, revealing 75%–100% of the clinical crown and only the interdental papilla, which is consistent with other researchers' findings (Melo et al. 2020; Desai et al. 2009; Nold et al. 2014; Maulik and Nanda 2007). Smiles showing more than 2‐3 mm or over 3 mm of gingival tissue are considered less attractive (Calçada et al. 2014). The females in the present study presented a greater prevalence of high smile lines, whereas the males presented more medium smile lines, which is consistent with the findings of other studies (Melo et al. 2020; Nold et al. 2014; Al‐Johany et al. 2011). In our study, the percentage of high smile lines was 12% in individuals over 30 years of age and 26.31% in those under 30 years of age, indicating that with age, supporting tissues weaken, and the lip decreases. Smile width was up to the first molar in most individuals, with 87% of cases ranging between the second premolar and first molar, supporting findings from other studies, although one study reported the first premolar as most frequent, reflecting population differences (Melo et al. 2020; Al‐Johany et al. 2011),. Tooth shape was primarily square and then oval, similar to other studies reporting oval shape as most frequent, reflecting population differences (Melo et al. 2020; Brunetto et al. 2011). In males, triangular and oval teeth were more common than in females, who had more square teeth. Brunetto et al. reported similar results, with males having more square teeth and females having more triangular teeth (Brunetto et al. 2011). In contrast, Anderson et al. reported that square teeth were more common in males, reflecting population differences. Among specialists, opinions vary on the most attractive incisor shape, with orthodontists and restorative specialists preferring round‐squares and round shapes for females, respectively (Anderson et al. 2005). In uncertain cases, Nold et al. recommend selecting oval central incisors, as they are more likely to correlate with natural teeth than other shapes are, regardless of the patient's sex (Nold et al. 2014). The width‐to‐length ratio of teeth in our study was 73.20% of the golden ratio of 1.618, a finding that is consistent with other studies (Akl et al. 2022; Mosomi et al. 2024). However, a study in an English population suggested modified ratios of 71% for incisors and 61% for canines, which aligns with our findings (Kalia 2020).

Several studies in different populations have revealed notable variations in facial proportions and midline alignments. For instance, in a North Indian population, Khan et al. (Khan et al. 2016). reported that the majority of attractive females exhibited several facial ratios close to the golden ratio, whereas the corresponding values in males deviated more from this ideal. Similarly, Farkas et al. (Farkas et al. 2005). conducted an international anthropometric study and demonstrated significant racial differences in facial morphology among Americans, African‐Americans, Caucasians, Malaysians, Indians, Arabs, and Chinese. Moreover, Musa et al. (Musa et al. 2023). found that ethnic background influences the perception of midline deviations, with different thresholds for acceptability noted among Chinese and Black raters. These findings, when compared with our results, underscore the importance of considering population‐specific norms in facial and dental midline assessments. Incorporating such cross‐population comparisons provides a broader context for understanding the variations observed in our study and supports the notion that while aesthetic criteria are universally valued, subtle anthropometric differences exist between races and genders.

## 5 Conclusion

In our study, males' lower facial regions and females' upper facial regions occupied a larger portion of the vertical dimension, whereas females' eye and mouth widths occupied a smaller portion of the transverse dimension than males did. The order of landmarks relative to the facial midline from left to right includes the following: nasion, nose tip, philtrum, mouth midline, and dental midline. The studied population has a consonant smile arc, an upwards upper lip curvature, a medium smile line, with exposure up to the first molar, and square‐shaped teeth. Integrating these aesthetic standards into treatments ensures satisfactory and predictable outcomes. These results can be used at macroesthetic, miniesthetic and microesthetic levels by maxillofacial surgeons, prosthodontists, orthodontists, and restorative specialists. According to the impact of racial, sex, and age‐related factors on these parameters, similar studies in different populations are recommended for more accurate and aesthetically pleasing treatments tailored to each community.

## Author Contributions

**Dr. Zahra Bagheri** contributed to the study design, critical revision of the manuscript, interpretation of data, data collection, and approved the final version. **Dr. Vahid Mollabashi** was involved in critical revision, interpretation of data, data collection, and approved the final version. **Dr. Mohammad Mahdi Maleki** conceptualized and designed the study, drafted the original manuscript, contributed to critical revision, interpretation of data, data collection, and approved the final version. **Dr. Behnaz Alafchi** performed the statistical analysis, contributed to critical revision, interpretation of data, drafted the original manuscript, and approved the final version. All authors meet the authorship criteria, take full responsibility for their contributions, and agree to be accountable for all aspects of the work.

## Ethics Statement

This study adheres to the ethical and legal considerations of research, follows the principles and guidelines of the International Committee on Publication Ethics (COPE) and is approved by the Research Ethics Committees of Hamadan University of Medical Sciences with ethics code IR.UMSHA.REC.1402.601, and informed consent was obtained from all participants.

## Consent

All of the authors of this paper affirm that it has been submitted solely to this journal and that it is not concurrently under consideration for publication in another journal. All of the named authors have been involved in the work leading to the publication of the paper and have read the paper before it is submitted for publication.

## Conflicts of Interest

The authors declare no conflicts of interest.

## Data Availability

The data that support the findings of this study are available on request from the corresponding author. The data are not publicly available due to privacy or ethical restrictions.
